# Intracellular Crosstalk Between NMDA and Sigma-1 Receptors: A Novel Mechanistic Insight Relevant to Neuropsychiatric Pharmacotherapy

**DOI:** 10.3390/cells15181689

**Published:** 2026-09-17

**Authors:** Katarzyna Lipke, Agnieszka Piwowar

**Affiliations:** Department of Toxicology, Faculty of Pharmacy, Wroclaw Medical University, 50-367 Wroclaw, Poland; agnieszka.piwowar@umw.edu.pl

**Keywords:** sigma-1 receptor, NMDA receptor, receptor crosstalk, neuroprotection, excitotoxicity, neuropsychiatric disorders

## Abstract

**Highlights:**

**What are the main findings?**

• The σ1R and NMDAR are functionally linked through shared intracellular signaling pathways.
• Many pharmacological (e.g., ketamine, dextromethorphan) and physiological agents (neurosteroids) act through both σ1R and NMDAR mechanisms.

**What are the implications of the main findings?**

• σ1R–NMDAR dysregulation may contribute to psychiatric and neurodegenerative disorders.
• The σ1R–NMDAR axis represents a promising mechanistic therapeutic target.

**Abstract:**

Formerly recognized as an opioid receptor, the sigma-1 receptor (σ1R) is a multifunctional chaperone protein that plays a crucial role in regulating neuronal signaling, neuroprotection, and synaptic plasticity. Increasing evidence highlights a tight functional association between the σ1R and the N-methyl-D-aspartate receptor (NMDAR), a central mediator of excitatory neurotransmission and calcium-dependent neuronal processes. This review summarizes the shared signaling pathways underlying σ1R and NMDAR activity, including calcium homeostasis, calcium/calmodulin-dependent protein kinases (CaMKs), protein kinase C (PKC), phosphoinositide 3-kinase/protein kinase B (PI3K/Akt), and mitogen-activated protein kinase (MAPK) cascades, as well as transcriptional regulators such as cAMP response element-binding protein (CREB), nuclear factor kappa-light-chain-enhancer of activated B cells (NF-κB), and B-cell lymphoma 2 (Bcl-2). Experimental data demonstrate that pharmacological agents initially characterized as NMDAR antagonists—such as ketamine, dextromethorphan, and memantine—also interact with σ1R, suggesting that their therapeutic efficacy may arise from coordinated modulation of both receptor systems. These findings collectively indicate that the σ1R is a key regulatory element enabling proper NMDAR function and that disruption of this interaction may contribute to excitatory imbalance implicated in the pathophysiology of neuropsychiatric disorders. Recognizing the σ1R–NMDAR crosstalk as an integrated signaling axis may thus inform the development of dual-target therapeutic strategies aimed at improving neuronal resilience and clinical outcomes in psychiatric and neurodegenerative diseases.

## 1. Introduction

One of the most prevalent and debilitating neuropsychiatric disorders worldwide, major depressive disorder (MDD), imposes a profound socioeconomic and healthcare burden [1,2]. Despite extensive research efforts, the incidence of depression continues to rise, highlighting the urgent need for more effective and individualized treatment strategies [3,4]. The pathophysiology of MDD is multifactorial, encompassing alterations in neurotransmission—particularly involving monoaminergic, GABAergic, and glutamatergic systems—as well as dysregulation of the hypothalamic–pituitary–adrenal axis, impaired brain-derived neurotrophic factor (BDNF) signaling, and perturbations within the microbiome–gut–brain axis [5,6,7,8]. These interconnected pathways underscore the complexity of depressive and other psychiatric disorders and suggest that targeting a single mechanism is unlikely to yield comprehensive therapeutic outcomes.

Conventional antidepressant treatments, primarily based on the monoamine neurotransmitter theory, aim to restore serotonergic, noradrenergic, or dopaminergic balance [9]. However, their limited efficacy, delayed onset of action, and considerable side-effect profiles have restricted their clinical success, with many patients failing to achieve full remission. This therapeutic gap has stimulated the exploration of alternative molecular targets and mechanisms underlying depressive symptoms. Notably, the emergence of N-methyl-D-aspartate receptor (NMDAR) antagonists, such as ketamine and its derivatives, has revolutionized the conceptual framework of antidepressant therapy by demonstrating rapid and sustained antidepressant effects through modulation of glutamatergic neurotransmission and synaptic plasticity [10,11].

Increasing attention has turned to the sigma-1 receptor (σ1R), a unique intracellular chaperone protein implicated in neuroprotection, neuroplasticity, and the regulation of multiple neurotransmitter systems. Activation of the σ1R has been shown to modulate calcium signaling, BDNF expression, and cellular resilience under cellular stress conditions, positioning it as a promising therapeutic target for neuropsychiatric disorders [12,13]. Integrating molecular insights into the complex network of neurochemical, endocrine, and immune interactions provides an essential foundation for the development of precise, mechanism-based treatment strategies. Such an approach may ultimately lead to more effective interventions for patients suffering from MDD and related conditions. Furthermore, the mutual cellular signaling crosstalk between the σ1R and NMDAR remains insufficiently characterized. Understanding this molecular interaction may redefine current paradigms of rapid-acting antidepressant mechanisms.

The objective of this review is to provide a comprehensive analysis of the signaling interplay between the σ1R receptor and the NMDAR in neuronal cells with potential implications for mechanistic relevance in neuropsychiatric pharmacology. Since the clinical data are limited, a structured literature search was conducted with a focus on manual screening of the literature. The literature search incorporated both Medical Subject Headings (MeSHs) and free-text keywords, which were combined using the Boolean operators AND and OR.

A thorough PubMed search was performed. The first step was an overall search with the following query: “sigma 1 receptor”[Supplementary Concept] OR “sigma 1 receptor”[Title/Abstract] OR “sigma 1 receptor”[Title/Abstract] OR “oprs1 protein human”[Supplementary Concept] OR “oprs1 protein human”[Title/Abstract] OR “sigma 1 receptor”[MeSH Terms] OR (“sigma 1”[All Fields] AND “receptor”[Title/Abstract]). In the screening, the literature related to the NMDAR was selected. Terms such as “NMDA”, “NMDAR”, and “glutamate”, among others, served as the inclusion criteria. The articles relating to the oncology, radioligands and synthesis of new compounds were mostly excluded as they were considered outside the scope. From the retrieved results, studies providing detailed insights into mechanistic cellular signaling pathways were selected. The review included only full-text articles published in peer-reviewed journals. Studies that did not address the objectives of the review and conference abstracts lacking an associated full-text publication were excluded. Given the heterogeneity of the included study designs, a quantitative synthesis was not feasible. Accordingly, the available evidence was synthesized qualitatively and structured into thematic categories. This approach facilitated a comprehensive synthesis and critical evaluation of the available evidence while also identifying existing knowledge gaps and areas requiring further research.

Data acquisition was conducted as a narrative review; in several instances, additional relevant articles were identified through the reference screening lists of the initially retrieved publications.

## 2. σ1R Receptor

The σ1R is a unique ligand-operated molecular chaperone that differs structurally and functionally from classical neurotransmitter receptors [14]. Although it was originally proposed to be a novel opioid receptor, it is now recognized primarily as a molecular chaperone. Nevertheless, the term “receptor” has been retained for historical reasons and reflects its capacity to bind a diverse range of ligands.

When inactive, the σ1R forms complexes with binding immunoglobulin protein (BiP), and calcium depletion from the endoplasmic reticulum (ER) leads to the dissociation of this complex [14,15]. Its ability to bind a diverse range of endogenous and exogenous ligands—including neurosteroids, psychostimulants, and certain antidepressants—reflects its versatile regulatory role in cellular signaling [15]. The σ1R is widely distributed throughout the central nervous system (CNS), with particularly high concentrations in the hippocampus, cerebral cortex, cerebellum, and motor neurons of the spinal cord [16]. This enrichment in key brain regions involved in cognition, emotion, and motor control highlights its relevance to both normal neuronal function and neuropsychiatric disease mechanisms [17]. Beyond the CNS, σ1R expression has also been observed in peripheral tissues such as the liver, heart, and immune cells, indicating its systemic physiological significance [18].

**Figure 1:**
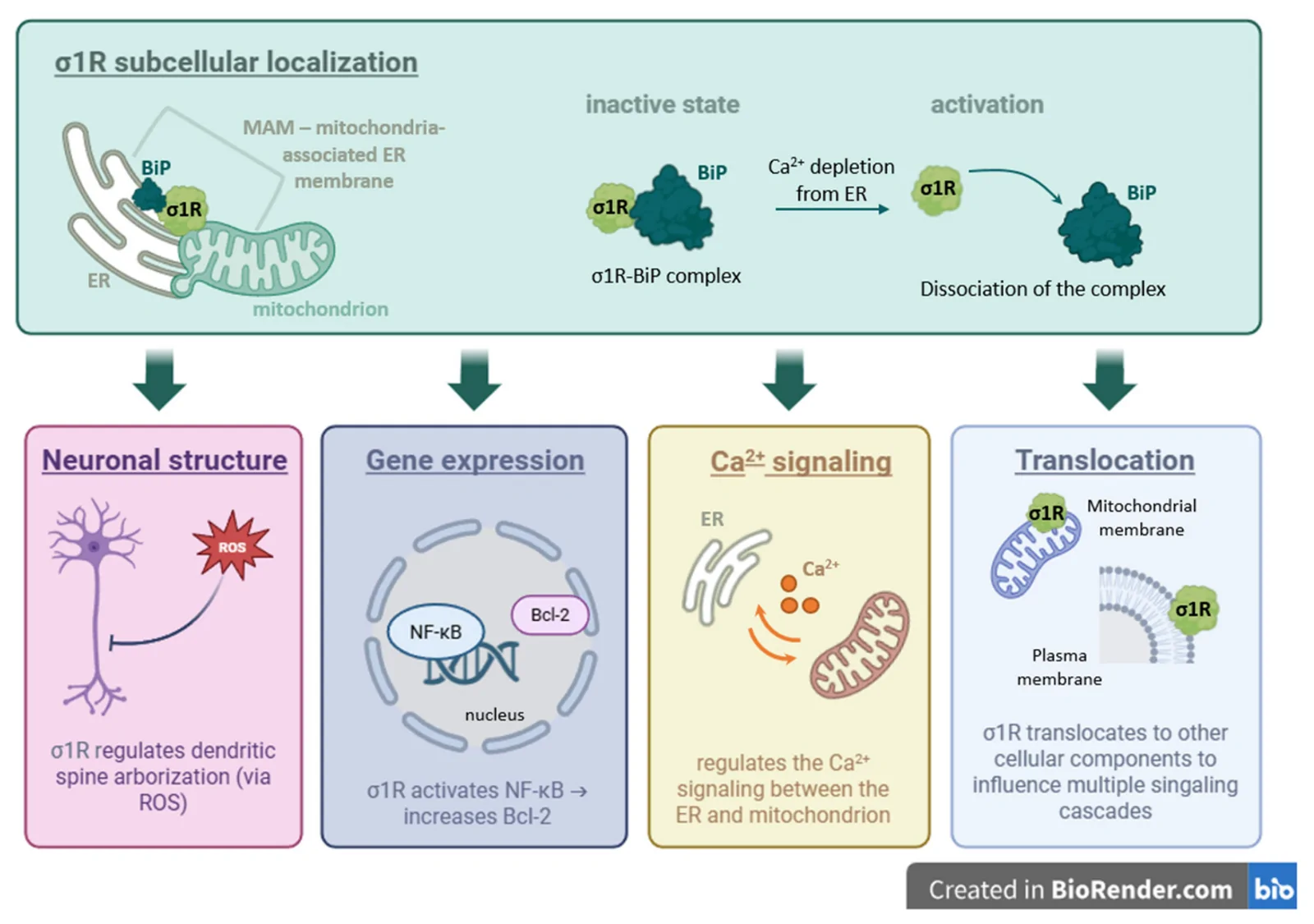
The schematic representation of the σ1R localizations and signaling pathways. When inactive, the σ1R remains bound to the binding immunoglobulin protein (BiP) at the endoplasmic reticulum (ER). Upon activation, the σ1R dissociates from BiP and modulates Ca^2+^ exchange between the ER and mitochondria. Activated σ1R can also stimulate the NF-κB pathway, leading to increased Bcl-2 mRNA expression, and promotes dendritic spine arborization. Moreover, the σ1R regulates reactive oxygen species (ROS) levels and, upon translocation to the plasma membrane, modulates ion channels and other membrane-associated receptors.

At the subcellular level, the σ1R is predominantly localized to the ER, where it is concentrated at the mitochondria-associated ER membrane (MAM)—a critical interface for calcium exchange, lipid metabolism, and cellular stress signaling [19]. The σ1R regulates the Ca^2+^ signaling between the ER and mitochondrion [20,21]. The σ1R is also responsible for controlling dendritic spine arborization in neurons via the regulation of the level of reactive oxygen species (ROS) at the ER [20,22]. Furthermore, the σ1R controls gene expression of an antiapoptotic protein, B-cell lymphoma 2 (Bcl-2), by activating nuclear factor kappa-light-chain-enhancer of activated B cells (NF-κB) [20,23]. Figure 1 presents the summarized visual presentation of σ1R localizations and signaling pathways.

Emerging evidence indicates that the σ1R exerts potent neuroprotective effects by mitigating oxidative stress, promoting mitochondrial integrity, and facilitating neurotrophic signaling, particularly through the modulation of BDNF pathways [24,25,26,27,28,29,30]. Consequently, σ1R dysfunction has been implicated in the pathogenesis of several neurodegenerative and neuropsychiatric disorders, including Alzheimer’s disease, Parkinson’s disease, amyotrophic lateral sclerosis, and MDD [17,31,32,33,34,35]. Therefore, there is an ongoing search for a σ1R-targeted therapeutic intervention [31,32,36,37]. These findings collectively position the σ1R as a promising molecular target for therapeutic strategies aimed at neuroprotection and functional restoration of neural circuits.

## 3. NMDAR

The NMDAR is a glutamate-gated ion channel that plays a pivotal role in excitatory neurotransmission, synaptic plasticity, and cognitive processes such as learning and memory [38]. Structurally, the NMDAR is a heterotetrameric complex typically composed of two obligatory NR1 (GluN1) subunits and two NR2 (GluN2, A–D) or, less frequently, NR3 (GluN3, A–B) subunits. The functional receptor requires simultaneous binding of glutamate and the co-agonist glycine or D-serine, as well as depolarization to relieve the magnesium ion (Mg^2+^) block, thus permitting calcium and sodium influx and potassium efflux [39].

**Figure 2:**
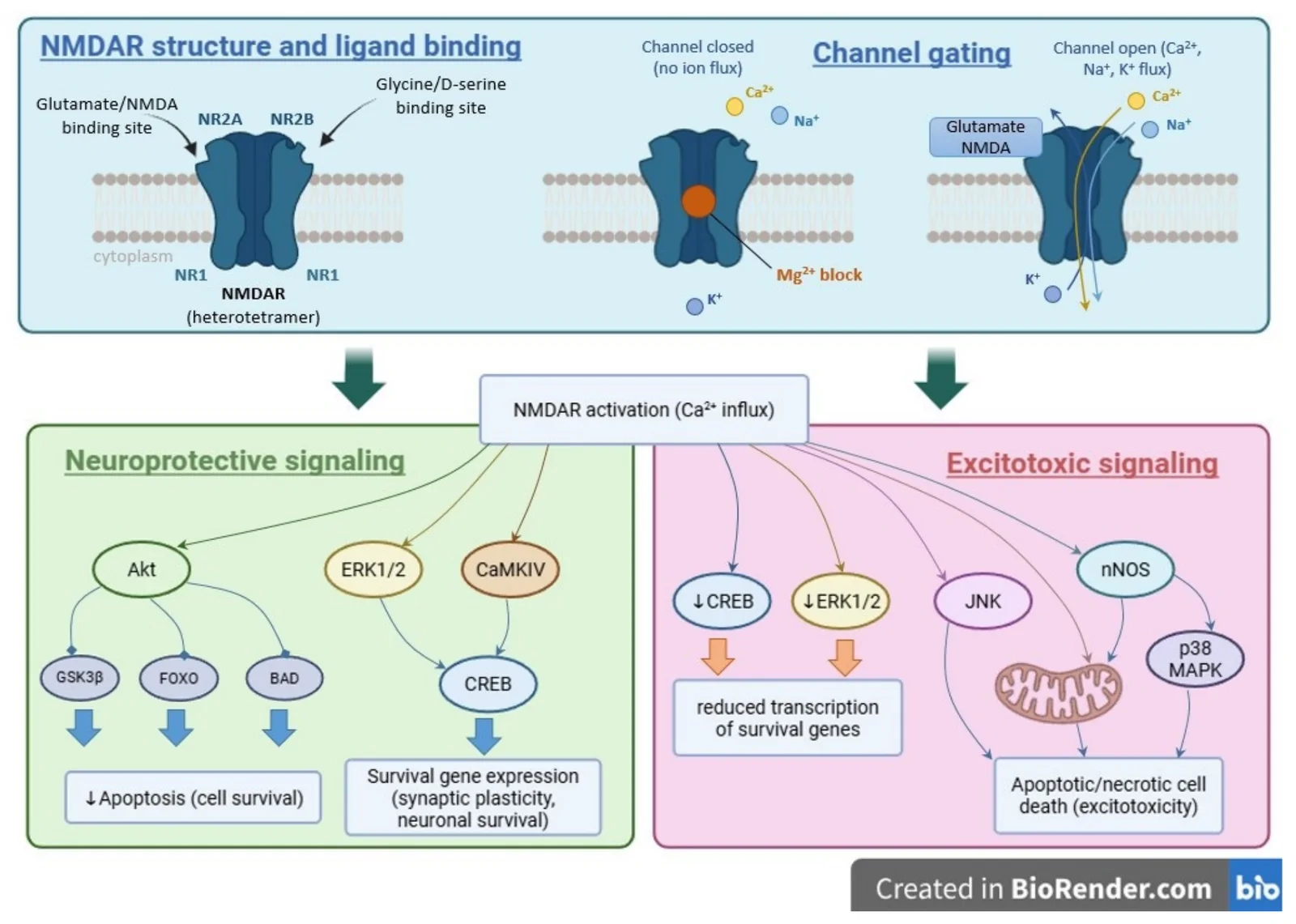
Structure of the NMDAR and signaling pathways involved with its activation: neuroprotective signaling and excitotoxic signaling. The pro-survival effects of NMDAR activation are mediated through the stimulation of the Akt pathway, leading to inhibition of GSK3β, FOXO, and BAD, thereby preventing apoptotic cell death. Additionally, NMDAR signaling engages ERK1/2 and CaMKIV to promote CREB phosphorylation and transcriptional activation of survival genes. Conversely, pro-death signaling is characterized by downregulation of CREB and ERK1/2, loss of mitochondrial membrane potential, and activation of stress-associated pathways including JNK, nNOS, and p38 MAPK.

NMDAR is widely distributed throughout the CNS, with the highest densities in the hippocampus, prefrontal cortex, and cerebral cortex—regions crucial for cognition, emotional regulation, and synaptic integration [40]. At the cellular level, the NMDAR is predominantly located at postsynaptic densities of excitatory synapses, but extrasynaptic populations are also present and serve distinct signaling roles [41]. Synaptic NMDAR activation promotes neuroplasticity and cell survival pathways through calcium-dependent activation of calcium/calmodulin-dependent protein kinase II (CaMKII), cAMP response element-binding protein (CREB), and BDNF signaling, whereas excessive or prolonged activation, particularly of extrasynaptic receptors, triggers excitotoxic response cascades leading to oxidative stress, mitochondrial dysfunction, and neuronal death [42,43]. The schematic presentation of NMDAR signaling pathways is shown in Figure 2.

Dysregulation of NMDAR function has been implicated in the pathophysiology of numerous neuropsychiatric and neurodegenerative disorders, including MDD, schizophrenia, Alzheimer’s disease, and ischemic stroke [44]. In depression, altered glutamatergic transmission and NMDAR overactivity contribute to synaptic deficits and neuronal atrophy. The discovery of rapid-acting antidepressant effects of NMDAR antagonists, such as ketamine and its metabolite (2R,6R)-hydroxynorketamine, has reshaped therapeutic paradigms by demonstrating that transient NMDAR inhibition can restore synaptic connectivity and induce rapid neuroplastic adaptations [45]. On the other hand, complete blockage of the NMDAR also results in disruption of the neuronal plasticity [46]. Beyond psychiatry, NMDAR modulation holds therapeutic promise for limiting excitotoxic damage in neurodegeneration and ischemia [47]. The multifaceted roles of the NMDAR in neuronal signaling, plasticity, and survival make it a central molecular target for developing precise treatments in neuropsychiatric medicine.

## 4. σ1R and NMDAR Crosstalk

**Figure 3:**
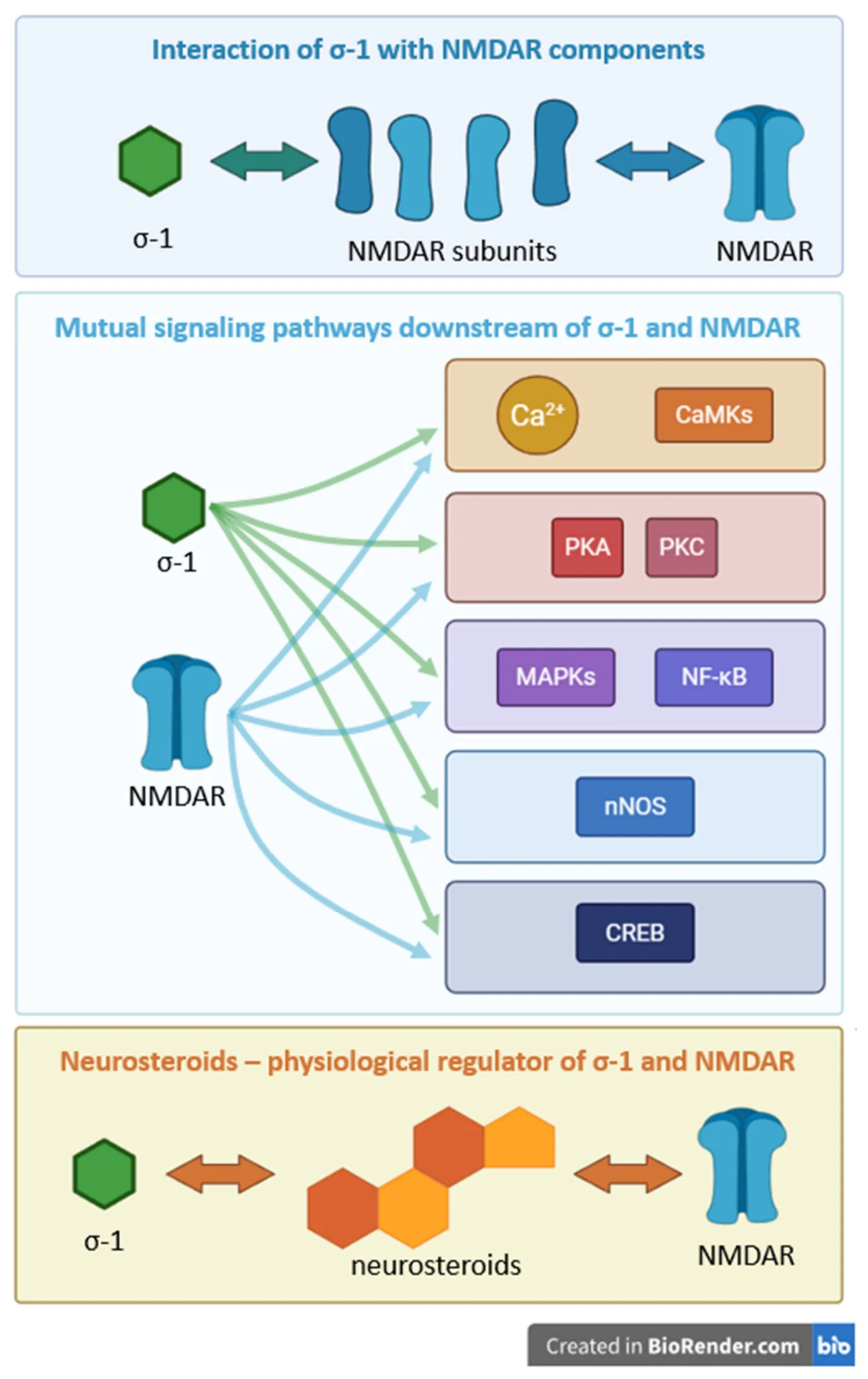
A graphical summary of the concepts addressed in Section 4.

The functional interplay between the σ1R and the NMDAR represents a crucial mechanism in regulating neuronal excitability, calcium homeostasis, and synaptic plasticity. Both receptors are highly expressed in the CNS and are intimately involved in processes that determine neuronal survival or degeneration under physiological and pathological conditions. While the NMDAR mediates excitatory neurotransmission through glutamate-dependent calcium influx, the σ1R acts as a ligand-regulated chaperone protein that fine-tunes cellular responses to stress conditions by modulating ion channel activity, protein folding, and intracellular signaling cascades. Their interaction forms a dynamic regulatory network that maintains the delicate balance between neuroprotection and excitotoxicity. Figure 3 provides a visual presentation of the key concepts discussed in the following section.

### 4.1. Interaction of σ1R with NMDAR Subunits

**Figure 4:**
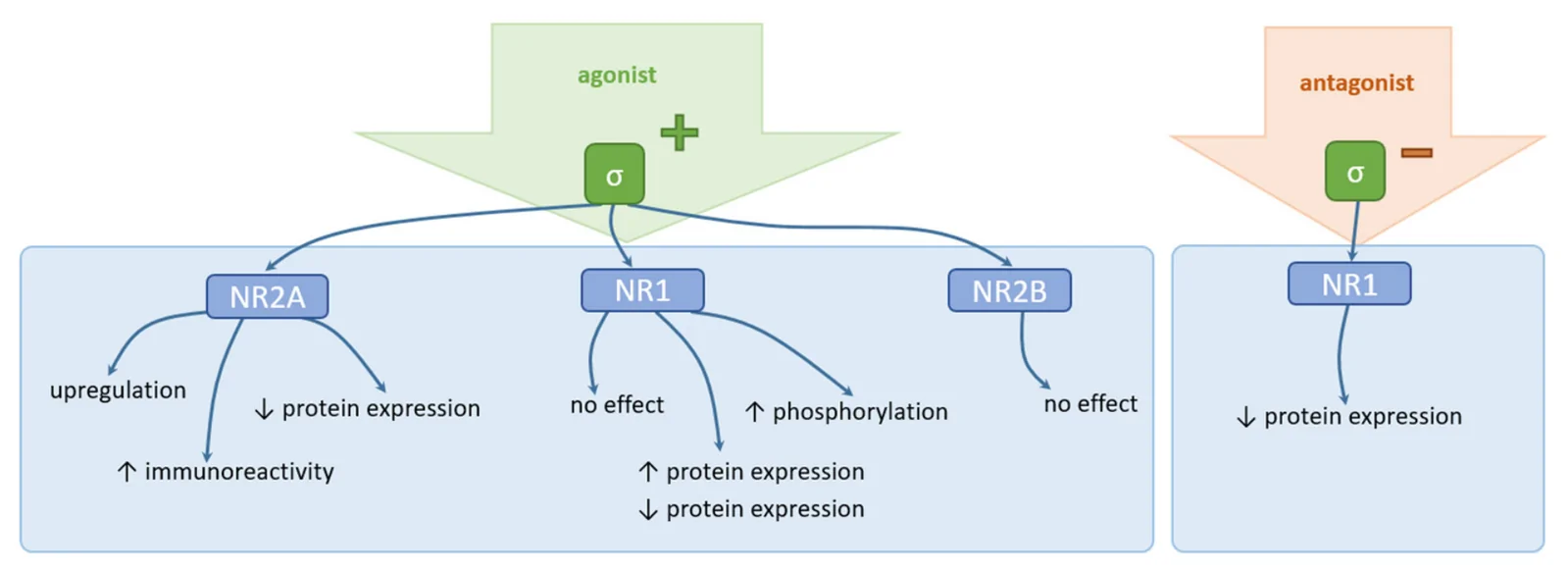
The schematic visualization of the interaction between σ1R and NMDAR subunits.

Upon activation, the σ1R dissociates from its inhibitory binding protein, BiP, and interacts with various membrane and cytosolic proteins. One of its key targets is the NMDAR complex, mainly the NR1 subunit. Evidence from biochemical and electrophysiological studies demonstrates that the σ1R can physically associate with NMDAR subunits, thereby modulating receptor trafficking to the cell surface and functional responsiveness [48,49]. Through this interaction, the σ1R can either potentiate or dampen NMDAR-mediated currents depending on cellular context, ligand type, and neuronal activity state. Upon stimulation with a σ1R agonist, NR1 subunit expression and phosphorylation can be variably modulated, resulting in either upregulation [50,51,52,53,54], downregulation [55], or no detectable effect [56,57]. Comparable variability is observed for NR2A, where σ1R agonists may enhance immunoreactivity and protein levels or, alternatively, reduce expression [55,58,59,60]. In contrast, NR2B appears largely unaffected by σ1R activation [55]. Furthermore, σ1R antagonists have been reported to decrease NR1 protein expression, highlighting the modulatory influence of the σ1R on specific NMDAR subunits [61]. A summary of the potential effects of σ1R modulation on NMDAR subunits is presented in Figure 4.

The demonstrated physical interaction between σ1R and NMDAR subunits provides compelling evidence for their functional interdependence. This molecular association suggests that the σ1R exerts its modulatory effects on neuronal excitability and survival at least in part through direct regulation of NMDAR activity.

### 4.2. Mutual Downstream Pathways of σ1R and NMDAR Activation

The σ1R and NMDAR share several convergent intracellular signaling pathways that underlie their roles in neuronal survival and plasticity. Several studies indicate that σ1R activation can influence intracellular calcium dynamics through multiple mechanisms. Specifically, σ1R agonists have been shown to elevate intracellular Ca^2+^ levels via indirect activation of protein kinase C (PKC) [62], suggesting a role in calcium-dependent signaling modulation. Interestingly, the σ1R agonist (+)-SKF-10,047 was also reported to inhibit NMDA-induced Ca^2+^ influx in primary neurons [38,63], implying that the σ1R may act as a regulatory buffer under conditions of excessive excitatory stimulation. Furthermore, evidence that Ca^2+^ entry through the NMDAR is required for σ1R agonists to modulate NMDAR-mediated currents [64] supports the concept of a bidirectional feedback mechanism. This reciprocal interaction suggests that the σ1R not only modulates NMDAR function but also relies on NMDAR-mediated calcium influx for its own regulatory activation, establishing a dynamic interplay that stabilizes neuronal excitability.

Another important element of calcium-dependent signaling involves calcium/calmodulin-dependent protein kinases (CaMKs), which play key roles in synaptic plasticity and neuronal survival. It was demonstrated that antagonism of the σ1R reduces the levels of phosphorylated CaMKII [65], indicating that σ1R activity contributes to maintaining CaMKII signaling. Consistently, σ1R antagonists were also shown to diminish NMDA-induced phosphorylation of CaMKII [66], further supporting the close functional association between the σ1R and NMDAR-mediated calcium signaling. In contrast, σ1R activation was reported to increase the expression of calcium/calmodulin-dependent protein kinase IV (CaMKIV) [67] and to restore CaMKIIα autophosphorylation [68], which is a critical process for sustaining long-term potentiation and synaptic strength.

σ1R signaling is closely associated with the regulation of key protein kinases involved in neuronal survival and plasticity. Activation of the σ1R promotes the translocation of pan-PKC from the cytosol to the plasma membrane, resulting in enhanced enzymatic activity [50]. This mechanism parallels NMDAR-mediated calcium influx, which similarly activates PKC to regulate receptor trafficking and synaptic plasticity [69]. Both PKC and protein kinase A (PKA) have been implicated in σ1R-dependent upregulation of the NMDAR NR1 subunit [52,54], suggesting that the σ1R modulates NMDAR expression and function through shared kinase-dependent signaling routes. In addition, the PI3K/Akt pathway—well-established as a downstream effector of NMDAR activation contributing to cell survival and synaptic strengthening—is also stimulated by the σ1R [43]. Specifically, σ1R agonists increase Akt phosphorylation via PI3K activation [70], leading to the restoration of CREB activation and BDNF expression [68,71], both key components of NMDAR-driven neuroprotective signaling.

Mitogen-activated protein kinases (MAPKs) constitute a critical downstream signaling pathway shared by both σ1R and NMDAR activation. Among them, ERK1/2 is well known for mediating transcriptional responses that promote neuronal survival and synaptic plasticity. Activation of the NMDAR induces ERK1/2 phosphorylation through Ca^2+^-dependent mechanisms, leading to the activation of CREB and the expression of neuroprotective genes such as BDNF [43,72]. Similarly, σ1R stimulation enhances ERK1/2 phosphorylation [73,74,75,76], indicating that both receptors converge on this MAPK-dependent pathway to facilitate neurotrophic signaling and neuronal resilience. However, σ1R-mediated modulation of MAPKs appears to be context-dependent. While ERK1/2 activation aligns with NMDAR-driven pro-survival effects, σ1R agonists have also been shown to increase p38 MAPK phosphorylation [77], a kinase commonly associated with stress and apoptotic responses. In contrast, σ1R antagonism reduces p38 phosphorylation [65], further underscoring the receptor’s regulatory influence over MAPK activity. Interestingly, the NMDAR can also engage the p38 pathway under excitotoxic conditions, contributing to neuronal injury when overstimulated [43].

Neuronal nitric oxide synthase (nNOS)–mediated nitric oxide (NO) production is a well-established component of NMDAR-dependent signaling, exerting dual roles in neuronal physiology and pathology. Under physiological conditions, moderate activation of the NMDAR stimulates nNOS through calcium/calmodulin interactions, contributing to CREB phosphorylation and synaptic plasticity. However, excessive NMDAR activation during excitotoxicity triggers overproduction of NO, leading to oxidative stress and neuronal injury [78,79]. Evidence indicates that the σ1R plays a modulatory role within this pathway. In σ1R knockout (σ1R^−^/^−^) mice, reduced NMDAR activity was accompanied by attenuated nNOS activation and decreased NO production, suggesting that the σ1R supports the coupling between NMDAR stimulation and downstream nNOS signaling [80]. Similarly, pharmacological inhibition of σ1R resulted in suppression of nNOS–NO–CREB signaling, an effect that could be reversed by NMDA administration, indicating that σ1R functionally contributes to the integrity of NMDAR-dependent signal transduction [81].

Glutamate-induced excitotoxicity is a well-characterized process driven primarily by NMDAR overactivation, leading to excessive intracellular calcium influx and the subsequent engagement of pro-apoptotic signaling cascades. One of the key pathways activated under these conditions is the NF-κB pathway, which mediates inflammatory and apoptotic gene expression in response to excitotoxic stress. Interestingly, the σ1R appears to exert a counter-regulatory influence on this process. While glutamate stimulation activates NF-κB, treatment with σ1R agonists effectively suppresses this activation, suggesting a modulatory role for σ1R in dampening NMDAR-driven neuroinflammatory signaling [82]. Beyond transcriptional regulation, σ1R agonists also mitigate downstream apoptotic mechanisms, as evidenced by their ability to reduce glutamate-induced caspase-3 activation in neuronal cultures [83]. In in vivo models of excitotoxic brain injury, σ1R activation similarly attenuates caspase-3 cleavage and DNA fragmentation, further supporting its anti-apoptotic potential [84]. Moreover, σ1R stimulation enhances the expression of the anti-apoptotic gene Bcl-2 following glutamate exposure, reinforcing neuronal resistance to excitotoxic stress [85].

**Figure 5:**
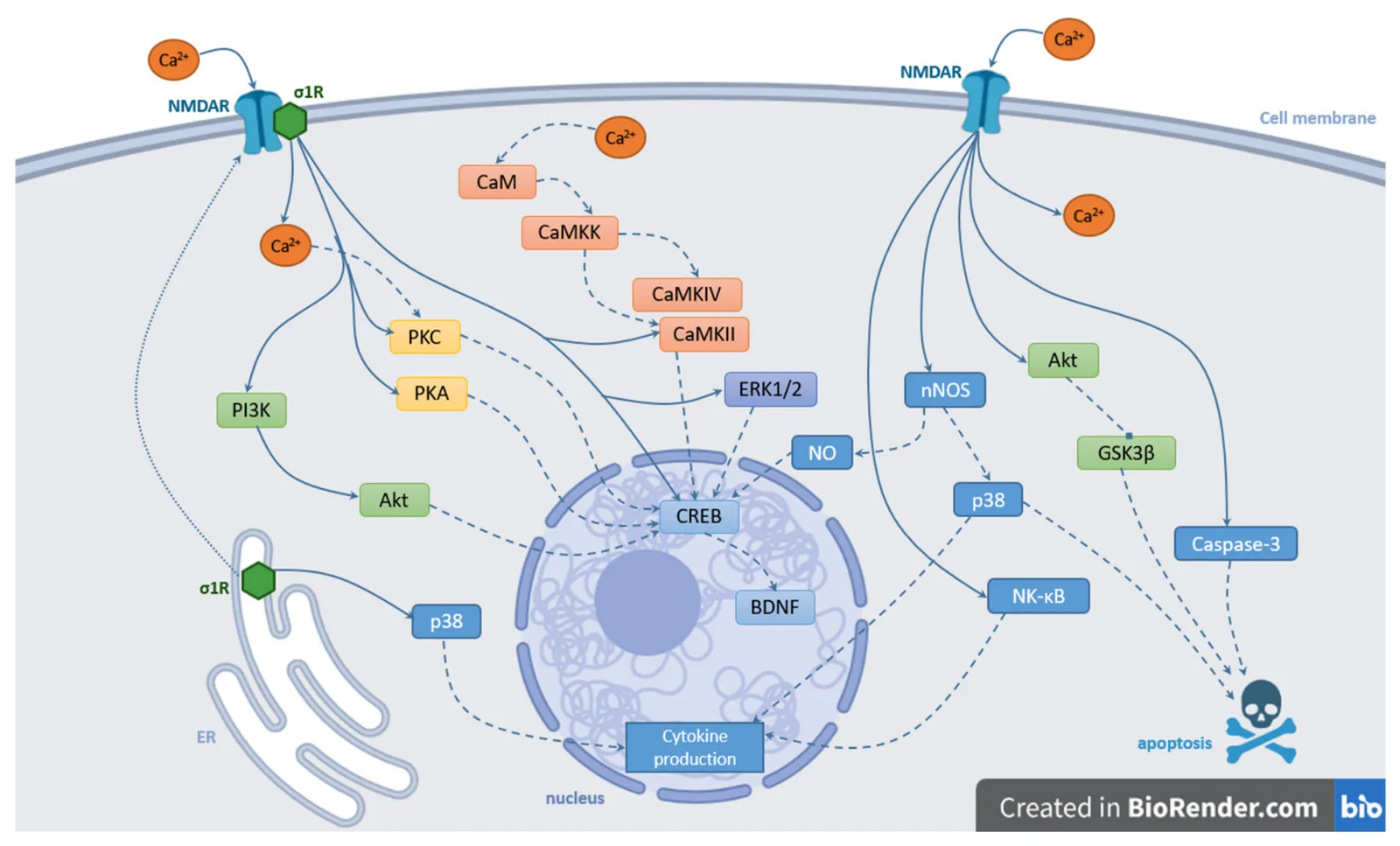
Proposed model of σ1R–NMDAR crosstalk in neuronal signaling and neuroprotection. Based on the current evidence, σ1R activity appears to be critical for promoting pro-survival NMDAR signaling, acting as a necessary modulator for proper glutamatergic function. Activation of the σ1R–NMDAR axis triggers the mobilization of multiple intracellular kinases, including PKC, PKA, PI3K/Akt, CaMKs, and ERK1/2, which collectively enhance CREB phosphorylation and upregulate BDNF expression, thereby supporting neuronal plasticity and survival. In contrast, unmodulated NMDAR activity can engage pro-apoptotic pathways, including excessive NO production via nNOS, activation of p38 MAPK, caspase-3, and other cell death mediators. In schematic representations, solid arrows indicate experimentally observed effects, while dashed arrows denote likely downstream signaling events inferred from molecular biology studies.

Collectively, the current evidence underscores a multifaceted interplay between σ1R and NMDAR signaling, converging on shared intracellular pathways that determine the neuronal fate under both physiological and stress conditions. The σ1R modulates NMDAR-dependent calcium influx, thereby shaping the activation of CaMKs and PKC, which regulate receptor trafficking, CREB phosphorylation, and synaptic plasticity. Parallel modulation of the PI3K/Akt pathway by the σ1R further supports neuronal survival through the restoration of CREB and BDNF expression. Similarly, σ1R activation enhances ERK phosphorylation within the MAPK cascade while limiting excessive p38 activation associated with cell death. In addition, the σ1R fine-tunes nNOS activity downstream of the NMDAR, preventing pathological NO overproduction during excitotoxicity. Through suppression of NMDAR-induced NF-κB activation and inhibition of caspase-3 cleavage, coupled with the upregulation of Bcl-2, the σ1R maintains a balance between excitatory signaling and neuroprotection. Altogether, the σ1R functions as a dynamic molecular modulator that stabilizes NMDAR signaling—amplifying its adaptive, plasticity-related outputs while counteracting pathways that drive inflammation and neuronal apoptosis. Figure 5 summarizes the proposed σ1R–NMDAR crosstalk model.

### 4.3. Neurosteroids

Neurosteroids constitute an important class of endogenous modulators that exert regulatory effects on both the σ1R and NMDAR, providing a physiological basis for the functional interplay between these two receptor systems. Several neurosteroids, including pregnenolone, dehydroepiandrosterone (DHEA), and progesterone, display high affinity for the σ1R, and therefore modulate such processes as synaptic plasticity and cellular resilience [52,86,87,88]. Scientific data confirm DHEA and pregnenolone (as well as their sulphate esters) act as a σ1R agonist [89,90,91]. However, there are also reports that pregnenolone interaction with the σ1R may be more complex, and this steroid can also function as an inverse agonist [92]. Conversely, progesterone is recognized as a σ1R antagonist [92].

Concurrently, many of these compounds also modulate NMDAR function—either potentiating receptor activity at low concentrations or exerting inhibitory effects under conditions of excessive stimulation—thereby maintaining excitatory balance and preventing glutamate-induced excitotoxicity [89,90,93,94]. Through this bidirectional regulation, neurosteroids can fine-tune calcium signaling, influence downstream kinases such as CaMKII and Akt, and enhance the expression of neurotrophic factors, including BDNF [70,89,91]. The convergent modulation of the σ1R and NMDAR by neurosteroids thus represents a critical physiological mechanism linking excitatory neurotransmission with intracellular survival pathways, underscoring their role as endogenous integrators of the σ1R–NMDAR crosstalk essential for neuronal homeostasis and cellular stress adaptation.

## 5. Pharmacological Approaches Linking σ1R and NMDAR Function

Pharmacological modulation of the NMDAR has long been regarded as a key strategy for mitigating excitotoxicity and re-establishing neuronal equilibrium. The pharmacological action of several clinically relevant compounds was originally attributed to their role as NMDAR antagonists; however, growing evidence indicates that many of these agents also interact with the σ1R. This dual activity positions them as an additional mechanistic link within the broader σ1R–NMDAR interrelation. Through concurrent modulation of both receptors, such compounds may not only suppress excessive calcium influx and excitotoxic signaling but also engage σ1R-dependent pathways that enhance neuroprotection and synaptic resilience. Consequently, the therapeutic efficacy observed for certain NMDAR antagonists in neuropsychiatric disorders—such as the rapid antidepressant effects of ketamine [92,95]—may, at least in part, arise from their ability to modulate σ1R activity, highlighting a crucial intersection between glutamatergic and σ1R-mediated signaling in neuropharmacology.

### 5.1. Neuroprotective Role of σ1R in NMDA and Glutamate-Induced Excitotoxicity

Given the recognition of excitotoxicity as a major contributing factor in neurodegenerative processes, considerable research has focused on identifying effective modulators of NMDAR activity. In light of the interdependence of the σ1R and NMDAR, recent studies focus on the aspect of σ1R modulation as it may be of great importance in designing pharmacological approaches. Numerous studies have demonstrated that σ1R agonists represent promising candidates for therapeutic intervention in excitotoxic conditions (Table 1).

Experimental findings consistently indicate that σ1R activation confers significant neuroprotection against NMDAR-mediated excitotoxicity, subsequent neuronal cell death, and also brain injury in vivo [102]. Consequently, σ1R-targeting compounds have emerged as potential drug candidates for mitigating excitotoxic damage. Studies referenced in Table 1 collectively establish a substantive link between σ1R activity and NMDAR regulation, highlighting their interplay as a crucial mechanism in neuronal survival.

### 5.2. NMDAR Antagonists

Dextrometorphan (DXM), initially characterized as an NMDAR antagonist, has revealed additional therapeutic potential in neurodegenerative and neuropsychiatric contexts, where σ1R–NMDAR interactions are increasingly recognized as crucial modulatory mechanisms. Beyond NMDAR antagonism, DXM exerts anticonvulsant and neuroprotective effects that are abolished by σ1R antagonists, indicating that its beneficial actions are also dependent on σ1R activation [103,104]. Electrophysiological recordings demonstrate that DXM reduces the peak amplitude of evoked excitatory postsynaptic currents (eEPSCs), an effect that parallels σ1R agonist activity [105]. Since eEPSCs reflect synaptic responses mediated predominantly by glutamatergic transmission, this finding suggests that DXM, through σ1R engagement, may stabilize NMDAR-mediated excitatory signaling and prevent pathological hyperexcitability while preserving physiological synaptic communication. Similarly, both DXM and σ1R agonists attenuate the expansion of the severely hypoperfused cortical area following ischemic insult, indicating convergent mechanisms of neurovascular protection [106]. Furthermore, DXM and σ1R agonists inhibit traumatic spreading depression and anoxic depolarization, whereas classical NMDAR antagonists lacking σ1R affinity fail to exert comparable effects, underscoring the functional significance of σ1R modulation in mediating neuroprotection [107,108]. Notably, blockade of the σ1R abolishes DXM-induced protection against anoxic depolarization, confirming the necessity of σ1R involvement [108]. Similarly, the analogue of DXM, dimemorfan, was observed to be effective in protecting against ischemic stroke through σ1R agonism [109]. In behavioral paradigms, DXM displays biphasic effects on locomotor activity in mice—stimulatory at low doses and inhibitory at higher doses—reflecting the complex interplay between NMDAR inhibition and σ1R activation [110].

Ketamine, a novel fast-acting antidepressant, was initially characterized as a non-competitive NMDAR antagonist; however, accumulating evidence indicates that the σ1R contributes significantly to its mechanism of action [111,112,113]. Moreover, co-administration of a σ1R agonist was shown to potentiate the antidepressant efficacy of ketamine, supporting the hypothesis that σ1R activation synergistically amplifies ketamine-induced neuroplastic and behavioral responses [112]. A recent study also demonstrated that ketamine can promote the transfer of the σ1R from astrocytes to neurons via exosomes. This discovery sheds new light on the interplay between the mechanism of action of ketamine and the role of the σ1R [114].

These findings suggest that the therapeutic profile of ketamine cannot be fully explained by NMDAR antagonism alone but rather reflects a dual mechanism involving σ1R modulation as a key determinant of its rapid and sustained antidepressant activity.

Phencyclidine (PCP), a well-known non-competitive NMDAR antagonist, elicits robust psychotomimetic and locomotor-stimulating effects that are often used to model schizophrenia-like behavior in rodents. Interestingly, these behavioral outcomes appear to be modulated by σ1R activity. Administration of a σ1R agonist effectively attenuates PCP-induced hyperlocomotion and impairment of latent learning, indicating that σ1R activation can counterbalance the excessive excitatory and dopaminergic signaling triggered by NMDAR blockade [115,116,117]. Moreover, chronic PCP treatment was shown to reduce σ1R immunoreactivity in the hippocampal CA1 region and dentate gyrus—areas critically involved in cognitive processing and synaptic plasticity [118]. This downregulation of σ1R expression may reflect a compensatory response to disrupted glutamatergic homeostasis, further emphasizing the functional interplay between the σ1R and NMDAR in maintaining neuronal excitation–inhibition balance and behavioral regulation. The detrimental pharmacological effects of PCP and the lack of neuroprotective effect might be a result of insufficient σ1R modulation.

Memantine, a low-affinity, uncompetitive NMDAR antagonist widely used in Alzheimer’s disease therapy, has been investigated for its potential interaction with the σ1R. Although binding studies revealed that memantine exhibits only weak affinity for σ1R (Ki ≈ 20 µM) [119], functional evidence suggests possible pharmacological relevance under certain conditions. It was reported that memantine antagonized the effects of a σ1R agonist and failed to show additive or synergistic activity when co-administered with another σ1R ligand, indicating a potential overlap in their mechanisms of action without mutual enhancement [120]. However, despite these observations, the contribution of the σ1R to memantine’s pharmacological effects remains ambiguous, with current data insufficient to determine whether σ1R interaction plays a meaningful role in its neuroprotective or therapeutic properties.

## 6. Conclusions

**Figure 6:**
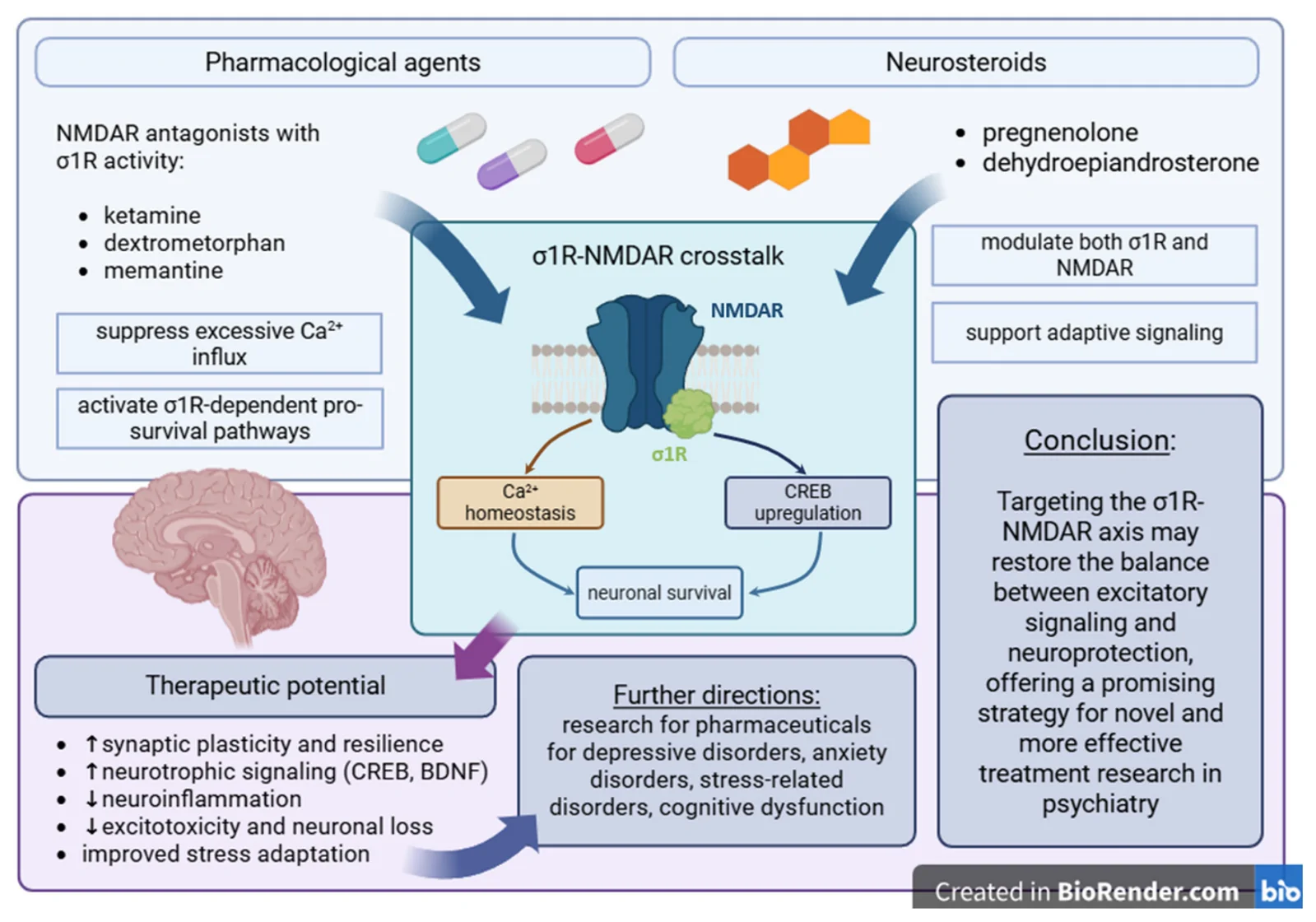
σ1R–NMDAR crosstalk and its potential therapeutic relevance. Functional interactions between the sigma-1 receptor (σ1R) and N-methyl-D-aspartate receptor (NMDAR) regulate Ca^2+^ signaling and promote neuroprotective pathways involving CREB supporting neuronal survival. Neurosteroids and pharmacological agents capable of modulating σ1R–NMDAR signaling may therefore shift this balance toward neuronal resilience. Targeting this functional axis represents a potential therapeutic strategy for neuropsychiatric disorders characterized by glutamatergic dysregulation, impaired neuroplasticity, neuroinflammation, or maladaptive stress responses.

Growing evidence indicates that the functional activity of the σ1R is closely and dynamically linked to NMDAR signaling. These two receptor signaling systems share numerous intracellular pathways—including calcium-dependent cascades, CaMKs, PKC, PI3K/Akt, MAPKs, and transcriptional regulators such as CREB and NF-κB—highlighting their coordinated role in maintaining neuronal excitability, synaptic plasticity, and cell survival. Pharmacological studies further support this interrelation, as several compounds initially identified as NMDAR antagonists, such as ketamine and DXM, also exhibit σ1R-mediated activity that may contribute to their therapeutic and neuroprotective effects. In parallel, neurosteroids act as endogenous ligands for both receptors, reinforcing the existence of a physiological σ1R–NMDAR interface essential for neuronal homeostasis. Taken together, these findings emphasize the importance of considering the σ1R–NMDAR interaction as a unified therapeutic target, particularly in neuropsychiatric disorders where excitatory–inhibitory imbalance and glutamatergic dysfunction are prominent. Moreover, it is plausible that σ1R activity enables the proper functional regulation of the NMDAR, and that σ1R dysregulation could contribute to the etiology or progression of neuropsychiatric diseases. Thus, future therapeutic strategies may benefit from a dual-target approach addressing both σ1R modulation and NMDAR signaling to achieve more precise and effective neuroprotective and antidepressant outcomes. In light of the growing problem of psychiatric morbidity and the limited effectiveness of conventional antidepressant pharmacotherapy, the development of a new strategy for designing pharmacological treatment is becoming increasingly important. The studies presented in this review suggest the significance of the σ1R–NMDAR relationship as a mechanistic target for the design of new therapeutics. Another review thoroughly describes the pharmacological approaches in affective disorders with a focus on σ1R modulation [121]. Despite the emerging literature reviews regarding this issue, we believe that the mechanistic approach to the σ1R–NMDAR is heavily underutilized in research and that the putting more focus on this angle would be greatly beneficial for future discoveries. A summary of the key points of this review is presented in Figure 6.

Although substantial evidence independently implicates σ1R and NMDAR signaling in neuropsychiatric disorders, direct clinical evidence demonstrating that their functional interaction determines treatment response remains limited. We propose that the σ1R acts as a context-dependent modulator of NMDAR signaling, potentially restraining excessive Ca^2+^-dependent excitotoxicity while preserving or facilitating NMDAR-dependent neuroplasticity and survival signaling. This framework may be particularly relevant to major depressive disorder, in which pharmacological agents with σ1R-NMDAR related actions provide an initial translational link between the two systems. Targeting this integrated receptor crosstalk, rather than individual signaling components, could offer a novel precision-based strategy for neuropsychiatric pharmacotherapy, particularly in treatment-resistant depression. The potential to develop more effective therapies for psychiatric disorders—particularly depression—underscores the significance of σ1R–NMDAR crosstalk as a promising target in the design of novel pharmacological strategies.

## 7. Limitations and Future Directions

Despite the care taken in preparing this review, there are several limitations. The most significant one is the potential subjective bias in selecting articles during screening. Furthermore, the chosen studies utilize different study designs and experimental models. This heterogeneity results in difficulties related to the proper interpretation and synthesis of the data. The experimental models are based primarily on animals; therefore, there is also a compatibility gap between the research result and potential clinical implications. Also, very little data address the sigma-2 receptor subtype; therefore; the focus was put on the σ1R subtype.

Although the majority of the studies are preclinical, there are emerging clinical data regarding the potential of σ1R–NMDAR in the fields of psychiatry and neurology [122,123].

We would like to highlight the mechanistic approach to the molecular basis of σ1R–NMDAR crosstalk as a promising target for both preclinical and clinical research. Defining these interactions offers potential strategies for the precise design of new pharmaceuticals. Furthermore, an interesting area of research is taking this crosstalk into account when explaining the mechanism of action of currently used drugs.

## Figures and Tables

**Table 1:** The summary of the effects of σ1R agonists against excitotoxic NMDAR stimulation.

Nr.	Pharmacological Intervention			Experimental Model	Observation (in Relation to the NMDAR Stimulation Alone)	Ref.
	σ1R Agonist	NMDAR Stimulation	Time of Incubation or Intervention			
1.	ANAVEX2-73	glutamate	24 h	oligodendroglia (neonatal rat forebrains)	reduced cell death	[96]
2.	PPBP	glutamate	24 h	primary hippocampal neurons (embryonic mice)	reduced cell death	[97]
3.	(+)-SKF10047	glutamate	4 days	retinal ganglion cells (rat)	reduced cell death	[83]
4.	SKF10047	4-AP	4 min after observed depolarization	cerebrocortical nerve terminals (rat)	reduced glutamate release	[98]
5.	SKF10047	NMDA	24 h	cortical neurons (brains of embryonic mice)	neuroprotection	[99]
6.	PRE-084	Ibotenate	24 h	primary hippocampal neurons (mice)	reduced brain injury	[84]
7.	Amido-1-piperizine 2	glutamate	24 h	primary cortical neurons (rat)	reduced cell death	[100]
8.	PPBP	glutamate	24 h	primary cortical neurons (rat)	reduced cell death	[85]
9.	(+)-SKF10047	NMDA	24 h	dopaminergic neurons (rat)	protective effect	[101]

Abbreviations: NMDAR, N-methyl-D-aspartate receptor; PPBP, 4-phenyl-1-(4-phenylbutyl) piperidine; 4-AP, 4-aminopyridine; NMDA, N-methyl-D-aspartate.

## Data Availability

No new data were created or analyzed in this study. Data sharing is not applicable.

## Abbreviations

The following abbreviations are used in this manuscript:

σ1R	sigma-1 receptor
4-AP	4-aminopyridine
Bcl-2	B-cell lymphoma 2
BDNF	brain-derived neurotrophic factor
BiP	binding immunoglobulin protein
CaMKII	calcium/calmodulin-dependent protein kinase II
CaMKIV	calcium/calmodulin-dependent protein kinase IV
CaMKs	calcium/calmodulin-dependent protein kinases
CNS	central nervous system
CREB	cAMP response element-binding
DHEA	dehydroepiandrosterone
DXM	dextrometorphan
eEPSCs	excitatory postsynaptic currents
ER	endoplasmic reticulum
MAM	mitochondria-associated ER membrane
MAPK	mitogen-activated protein kinase
MDD	major depressive disorder
NF-κB	nuclear factor kappa-light-chain-enhancer of activated B cells
NMDAR	N-methyl-D-aspartate receptor
nNOS	neuronal nitric oxide synthase
NO	nitric oxide
PCP	phencyclidine
PI3K/Akt	phosphoinositide 3-kinase/protein kinase B
PKA	protein kinase A
PKC	protein kinase C
PPBP	4-phenyl-1-(4-phenylbutyl) piperidine
ROS	reactive oxygen species
